# Diagnoses among long-term care recipients in Norway: a nation-wide registry study on prevalence and data completeness

**DOI:** 10.1177/14034948251339873

**Published:** 2025-05-21

**Authors:** Inger Johanne Bakken, Thomas Nordbø Heyeraas, Øystein Døhl, Siri Rostoft, Inger Ariansen, Lisa Victoria Burrell

**Affiliations:** 1 Department of Chronic Diseases, Norwegian Institute of Public Health, Oslo, Norway; 2 Department of Registry Quality, Norwegian Institute of Public Health, Trondheim, Norway; 3 Department of Finance, Trondheim Municipality, Trondheim, Norway; 4 Department of Neuromedicine and Movement Science, Faculty of Medicine and Health Sciences, Norwegian University of Science and Technology, Trondheim, Norway; 5 Department of Geriatric Medicine, Oslo University Hospital, Oslo, Norway; 6 Institute of Clinical Medicine, University of Oslo, Oslo, Norway; 7 Centre for Care Research, Norwegian University of Science and Technology, Gjøvik, Norway

**Keywords:** Long-term care services, diagnoses, registry data, epidemiology, long-term institutional stay

## Abstract

**Background and aims:**

The Norwegian Registry for Primary Health Care (NRPHC) is a mandatory register for all publicly funded primary healthcare services in Norway, including long-term care (LTC) services. The registry holds information on diagnoses for recipients of LTC services, but little is known regarding data quality and completeness.

**Methods:**

The study population consisted of all LTC recipients in Norway in 2023 (*N*=393,446). We investigated the proportion registered with any diagnosis in NRPHC LTC data. We compared diagnoses reported by LTC providers with diagnoses reported by general practitioners and by specialist health services. Additionally, we investigated the distribution of diagnoses by type of LTC provided. Diagnoses presumed common among recipients of LTC were investigated (diabetes, cardiovascular diseases, chronic obstructive lung diseases, cancer, mental disorders/substance use disorders, dementia, intellectual disability and hip fracture).

**Results:**

Diagnoses were reported by LTC providers for 51.4% of LTC recipients. Diagnoses were more often reported for users with more complex services (long-term institutional stay: 85.5%; assistive technology: 30.5%). Including data from general practitioners and specialist health services increased prevalence estimates compared with using NRPHC LTC data alone (dementia: 14.2% vs 8.2%; chronic obstructive lung diseases: 12.7% vs. 4.1%). Based on all available data, the prevalence of reported dementia among recipients of long-term institutional stay was 58.7%. The prevalence of reported substance use disorders among recipients of home-based services was 13.4%.

**Conclusions:**

**Data on diagnoses reported by LTC providers to the NRPHC are insufficient and should be supplemented with data from other sources.**

## Background

The World Health Organization defines long-term care (LTC) services as services that ensure that people can maintain a level of functional ability consistent with their basic rights and human dignity, and include diverse services ranging in scope and complexity [1]. In Norway, one third of healthcare costs are allocated to such services [2]. As in other western countries, Norwegian LTC services are experiencing increasing demands, as the population is ageing and more people have complex care needs [3, 4]. Moreover, new and younger user groups are increasingly seeking care in municipal LTC services [5]. The result is a strained service with insufficient resources and a lack of sufficient qualified personnel [6, 7]. To advance the development of sustainable LTC services, information on recipients of LCT services is needed to advise service development and policy.

The Norwegian Registry for Primary Health Care (NRPHC) is a mandatory register containing detailed longitudinal information on governmentally funded primary healthcare services in Norway from 2017 onwards [8]. The main objectives are to facilitate administration, management, economic evaluation and quality assurance, as well as to facilitate research on primary healthcare services [9]. At the time of writing, the NRPHC consists of two main pillars: (a) data on LTC services reported from the municipalities; and (b) data on consultations in general practices (GPs) and out-of-hours services reported by the providers of such services as part of the reimbursement system. Here, we refer to long-term care service data as NRPHC LTC data and reimbursement data as NRPHC GP data.

NRPHC LTC data hold individual-level information such as demographics, type and extent of services received, disability level and diagnoses [8, 10]. Diagnoses registered as part of LTC data must be considered as relevant for the service provided. Only diagnoses considered in the evaluation of the extent of services needed are included. The diagnoses stem from the patients’ meetings with general practitioners or with specialists in secondary care. Thus, the diagnostic information in NRPHC LTC originates from the same source as diagnoses in the NRPHC GP or in the mandatory registry for patient encounters in specialist health services (the Norwegian Patient Registry, NPR). The NPR holds information on all hospitalisations and outpatient visits in Norwegian public specialist healthcare services from 2008 onwards [5]. In contrast to data in the NRPHC GP and in the NPR, NRPHC LTC information goes through several manual steps before reaching the registry.

Having a clearly diagnosed illness is not a prerequisite for receiving LTC services, and services are often allocated based on disability level [11]. The underlying cause for the need of a LTC service should, in most cases, still be documented by a diagnosis. LTC services are often allocated to user groups with specific diagnoses, and healthcare costs differ extensively between different diagnostic groups [12, 13]. Political action plans, laws and recommendations may target specific diagnostic groups (i.e. adult day care services for people with dementia). Diagnostic information can, hence, aid policy development, service planning and management on both a local and national level.

Unfortunately, large-scale population-based studies on LTC services in Norway are limited, very few studies have utilised data from NRPHC LTC [11, 14, 15], and little is known regarding the data quality and completeness of diagnostic data. We aim to evaluate the completeness in reporting of diagnoses to NRPHC LTC by comparing with diagnostic information in the NRPHC GP and in the NPR. We start with an overall analysis of all diagnoses registered in NRPHC LTC, and progress to study specific diagnostic groups including registry data also from the NRPHC GP and the NPR. We also investigate the relationship between diagnoses and the type of LTC service provided. Thus, this study is an evaluation of the data quality but also identifies the frequency and distribution of common diagnoses among users of LTC services. This information will hopefully spur more research interest in using NRPHC LTC data, and guide researchers as to which register gives the most complete diagnostic information. We hope that this insight will facilitate further research, contributing to a better knowledge base for developing sustainable LTC services.

## Methods

### Study population and data sources

The study population consisted of all inhabitants in Norway registered in the NRPHC as recipients of LTC services in 2023.

For all individuals in this study population, we extracted data on diagnoses from three different sources through linking the personal identification number:

1. NRPHC LTC: Diagnoses reported from the municipal healthcare services;
2. NRPHC GP: Diagnoses reported from primary care physicians and out-of-hours service providers for consultations in general practice;
3. NPR: Diagnoses reported from specialist healthcare service providers for outpatient visits and hospitalisations.

Municipalities report diagnoses to NRPHC LTC as either International Statistical Classification of Diseases, 10th revision (ICD-10) or International Classification of Primary Care, 2nd edition (ICPC-2) codes, depending on whether the data originated from specialists or general practitioners. The NRPHC GP holds ICPC-2 diagnostic information while ICD-10 is used in the NPR.

Demographic data (year of birth and sex) were retrieved from the NRPHC’s copy of the National Population Registry.

### Handling of information on diagnoses

To reduce the complexity of the diagnostic information, we restricted analyses to non-communicable diseases by grouping ICD-10 and ICPC-2 codes [16]. This concept includes diabetes, cardiovascular diseases (CVD), chronic obstructive lung diseases (COPD), cancer, mental disorders, substance use disorders and hip fractures (see Nystad et al. for definitions [16]). We additionally included dementia (ICD-10: F00-F03, G30; ICPC-2: P70) and intellectual disability (ICD-10: F70-F79, Q90.9, Q93.4, Q93.5, F84.2; ICPC-2: P85) as separate groups given their assumed prevalence among LTC service recipients.

From NRPHC LTC, we included all diagnoses marked as ‘valid’ in the registry in the year of interest (2023).

For chronic diagnoses (COPD, dementia, diabetes and intellectual disability) we additionally extracted all data available from NRPHC GP (July 2017–2023) and for 10 years from the NPR (2014–2023). For CVD, cancer, substance use disorders, mental disorders and hip fractures we included data from the last 5 years (2019–2023) from both NRPHC GP and the NPR.

### Grouping of services

We grouped the 23 types of LTC services in NRPHC LTC into mutually exclusive groups ranging from the most to the least extensive and complex service groups: Long-term institutional stay, short-term institutional stay, sheltered housing (for health and care purposes), home-based services (home care and practical assistance), support services for informal carers, services for meaningful activity (leisure activities) and assistive technology, see Burrell et al. for details [10]. Individuals receiving multiple services were categorised to the most extensive and complex service group.

### Statistics

Data handling and analyses were conducted using R (version 4.1.3) [17]. The main packages used were DBI and odbc for data bases handling, data.table for data cleaning, linkage and aggregation.

### Ethics

This quality assurance analysis was conducted in accordance with the regulations of the NRPHC and NPR. The protocol was controlled and approved by the Department of Registry Quality’s legal adviser and director. The registries do not encompass information such as names or detailed addresses, and personal identification numbers used for linkage are encrypted. Only authors IJB and TNH had access to individual-level data.

## Results

### Study population

A total of 393,446 persons 0–104 years old were registered as recipients of LTC services in 2023 (57.4% women). The mean age was 64.5 years (standard deviation 24.9 years); median age was 74 years (inter quartile range 46–84). LTC recipients with more complex services were typically older than those with less extensive services (data not shown).

### Diagnoses in the NRPHC LTC

Nearly 1 million diagnoses were reported to NRPHC LTC for the study population, either as ICD-10 or as ICPC-2 codes. Less than 0.1% of diagnoses were not consistent with either diagnostic system. These were excluded from the data set.

Overall, at least one diagnosis was registered in the NRPHC LTC for 51.4% of all LTC recipients in 2023. We observed that diagnoses were considerably more often reported to NRPHC LTC for users with more complex services (Table I). For instance, 85.5% of long-term institutional stay recipients, compared with 30.5% of assistive technology recipients, were registered with diagnoses. The median number of diagnoses in NRPHC LTC was four for people with long-term institutional stay but considerably lower in the other service groups.

**Table I.** Recipients of long-term care services in Norway 2023 (*N*=393,446): Proportion registered in Norwegian Registry for Primary Health Care Long-Term Care Services (NRPHC LTC) data with any diagnosis, and median number of different diagnoses registered, by type of service.

	*N*	Proportion with any diagnosis (%)	Median number of diagnoses (IQR)
Long-term institutional stay	43,661	85.5	4 (2–7)
Short-term institutional stay	57,998	66.1	2 (0–6)
Sheltered housing	37,442	69.4	2 (0–5)
Home-based services	209,707	39.8	0 (0–2)
Support services for informal carers	13,958	53.0	1 (0–2)
Services for meaningful activity	11,577	35.9	0 (0–1)
Assistive technology	19,121	30.5	0 (0–1)

IQR: interquartile range.

### Overlap of diagnoses between the available sources of registry data

We investigated the overlap of diagnoses between the available sources of registry data. Without NPR data, 13.0% of cases of dementia, 28.1% of cases of cancer and 25.3% of cases of substance use disorders would not have been identified. Similarly, 28.4% of cases of mental disorders would not have been identified without data from NRPHC GP. Without data from NRPHC LTC, 9.7% of cases of intellectual disability and 7.7% of cases of dementia would not have been identified.

Table II shows in detail what the proportion of the LTC recipients with diagnoses would have been given the availability of data sources. The estimated prevalences would have been much lower with access to diagnoses from NRPHC LTC only (first row in the table). For instance, the prevalence estimate for substance use disorders using all registry data combined was 10.9%, compared with 2.9% if using NRPHC LCT data alone. Comparably, the prevalence estimate for substance use disorders with access to diagnoses from NPR and NRPHC GP, but not NRPHC LTC, would have been 10.4%, which is only slightly lower than the prevalence estimate using all three registries (10.9%). Similarly, the prevalence estimate for diabetes was 17.4% using all registry data combined compared with 7.0% using NRPHC LCT data alone.

**Table II.** Recipients of long-term care services in Norway 2023 (*N*=393,446): Prevalence estimates for selected diagnoses, by different combinations of available registry data.

Registry source	Dementia	Intellectual disability	COPD	Diabetes	Cancer	CVD	SUD	Other mental disorders	Hip fractures
LTC	8.2	5.1	4.1	7.0	6.9	17.7	2.9	10.1	3.3
LTC, GP	11.8	5.9	10.1	16.0	16.4	38.4	8.1	29.9	4.5
LTC, NPR	11.6	6.6	10.4	15.4	20.8	41.0	9.4	24.1	8.2
LTC, GP, NPR	13.5	6.9	12.7	17.4	22.8	47.5	10.9	33.6	8.4
GP	9.2	3.7	9.3	15.2	13.9	32.9	7.1	26.8	1.8
NPR	9.0	5.1	9.8	14.6	19.3	37.1	8.8	19.7	7.2
GP, NPR	12.5	6.2	12.4	17.2	21.7	45.4	10.4	31.5	7.5

LTC: Norwegian Registry for Primary Health Care long-term care services; GP: Norwegian Registry for Primary Health Care General Practice; NPR: Norwegian Patient Registry.

We used a lookback time of 10 years for conditions presumed chronic: dementia, intellectual disability, chronic obstructive disease (COPD) and diabetes, and 5 years for other diseases: cancer, cardiovascular disorders (CVD), substance use disorders (SUD), other mental disorders and hip fractures.

### Distribution of selected diagnoses by type of long-term care service

Finally, we investigated the distribution of selected diagnoses by type of LTC service provided (Table III) by using diagnostic data from all available registers. The proportion with dementia was considerably higher among people with long-term institutional stay than in the other groups, as expected. Substance use disorders were common among users of sheltered housing and also among those with home-based services (13.3% and 13.4%, respectively).

**Table III.** Recipients of long-term care services in Norway 2023 (*N*=393,446): Prevalence estimates for selected diagnoses, by type of service using all available registry data.

	*N*	Dementia	Intellectual disability	COPD	Diabetes	Cancer	CVD	SUD	Other mental disorders	Hip fractures
Long-term institutional stay	43,661	58.7	1.6	12.5	18.5	24.5	63.4	7.1	23.2	21.2
Short-term institutional stay	57,998	15.4	1.0	21.7	22.8	37.4	71.0	9.2	22.7	17.5
Sheltered housing	37,442	12.6	23.8	12.6	18.6	14.8	42.0	13.3	36.6	7.7
Home-based services	209,707	5.9	4.7	11.4	16.6	21.3	41.3	13.4	41.2	4.4
Support services for informal carers	13,958	2.0	36.4	1.9	6.2	2.8	9.7	1.5	17.0	0.6
Services for meaningful activity	11,577	6.8	16.6	3.9	8.3	6.4	18.1	4.6	35.9	1.1
Assistive technology	19,121	2.6	0.1	13.5	19.0	30.2	64.0	2.8	12.9	6.8

Data sources: Norwegian Registry for Primary Health Care long-term care services; Norwegian Registry for Primary Health Care General Practice; Norwegian Patient Registry.

We used a lookback time of 10 years for conditions presumed chronic: dementia, intellectual disability, chronic obstructive disease (COPD) and diabetes, and 5 years for other diseases: cancer, cardiovascular disorders (CVD), substance use disorders (SUD), other mental disorders and hip fractures.

## Discussion

The present study found that 51.4% of all LTC recipients in 2023 had a registered diagnosis in NRPHC LTC. Even though this is a slight increase from a 2017 study that reported that 44% of LTC recipients had a registered diagnosis [18], the completeness in reporting of diagnostic information to NRPHC LTC is still far from adequate. Completeness in diagnostic information varied substantially between different types of LTC services, and the completeness increased as the extent and complexity of services increased. Moreover, completeness varied somewhat between different diagnostic groups, but was most often higher in NRPHC GP and NPR than in NRPHC LTC.

### Completeness in diagnostic information

Despite the discouraging results in the present study, the apparent dose–response relationship between the completeness in diagnostic information and the complexity of LTC services is positive. People receiving more intense and extensive services more often have a registered diagnosis and have a higher number of registered diagnoses. This association can be due to higher requirements for documentation of care recipients’ health status and disorders when applying for these extensive and expensive services. In addition, these care recipients probably have a history of receiving less extensive services and have been in repeated contact with healthcare services. As a result, the probability that a diagnosis is registered in NRPHC LTC likely increases.

In addition to differences in completeness depending on the type of services received, we identified differences between different diagnostic groups. Whereas the completeness for cancer, substance use disorders and mental disorders was especially poor in NRPHC LTC compared with NRPHC GP and NPR, the completeness for dementia and intellectual disabilities was better. This is in line with the high political focus on users with dementia and intellectual disability in LTC services in Norway and other Nordic countries for the last decades [19–21], and these groups are prevalent recipients of LTC services.

Given the generally deficient reporting of diagnoses to NRPHC LTC identified in the present study, we recommend that future research, policy development and service planning that use data from NRPHC LTC do not solely rely on diagnostic information from this register. Such studies should supplement with diagnostic information from other sources, such as health registers, surveys or clinical assessments.

The moderate completeness in diagnostic information in NRPHC LTC probably stems from the manual processes of reporting diagnoses, first to the municipality, and thereafter the municipalities’ process for registering the diagnosis correctly. In addition, Norway’s 357 municipalities have no financial incentive to register LTC recipients’ diagnoses.

### Diagnostic groups in different long-term care services

The present study also identified differences in prevalence between different diagnoses and service types. CVD and mental disorders are relatively prevalent throughout all types of LTC services, as in the general population [16]. Given that previous studies have found a prevalence of depressive symptoms of 29% among older adults in nursing homes [22], we would in fact expect the prevalence of mental disorders to be even higher.

Substance use disorders and mental disorders are most prevalent among care recipients living in sheltered housing or receiving home-based services. Intellectual disabilities are also prevalent among those living in sheltered housing. These results align with the deinstitutionalisation of care to people with intellectual disabilities or mental health illness from the 1980s onwards, which has increased the number of younger LTC recipients with these disorders [5]. Moreover, the deinstitutionalisation can also be contributing to the high percentage of care recipients with intellectual disabilities who live at home and receive support services to their informal carers, as these support services are mostly given to parents of younger care recipients [15].

The present study found that 58.7% of care recipients living in long-term institutions had a registered dementia diagnosis when using data from all available sources combined. However, a previous study found that approximately 80% of elderly living in nursing homes had dementia [23], indicating that Norwegian health registers may not capture all incidents of dementia in Norwegian nursing homes. This underdiagnosis of dementia has also been reported in another recent study that investigated the completeness of dementia diagnoses registered in the NRPHC GP and the NPR by comparing with diagnoses set by physicians following diagnostic testing [24]. The clear underreporting of dementia can probably be explained by a lack of assessment of people living in nursing homes who already receive the highest level of care possible. Dementia diagnosed by the nursing home physician is usually not registered in NRPHC LTC, nor in NRPHC GP.

Despite the identification of several highly prevalent diagnoses among care recipients, NRPHC LTC does not specify whether these diagnoses have triggered the LTC services allocated to care recipients. Unfortunately, we cannot identify the causes behind the services provided to specific individuals through the data registered in NRPHC LTC. In general, services are allocated based on individual needs and preferences, and the disability level of care recipients highly influences service allocation [11]. We can assume that the registered diagnoses may be a contributing factor to the care recipients’ increased disability, but we cannot determine this association based on register data.

### Strengths and limitations

The present study yields valuable information to researchers and policy makers through identifying the deficiencies of diagnostic information in NRPHC LTC and advising the use of additional sources of diagnostic information. We hope to make register data from NRPHC LTC more known and more used. The present study includes all LTC recipients in Norway, and hence has no bias related to sex, age, socioeconomic status, or geographical location. This ensures total population coverage and high external validity.

The present study results must be interpreted considering limitations that apply to all register studies. None of the investigated data registers are safeguarded against errors, misclassifications or omissions, despite being systematically and regularly monitored, analysed and corrected [8]. In addition, there may be discrepancies between recorded service use and actual service use [25], and between registered diagnoses and actual diagnoses. However, a previous quality study indicated that the diagnostic information registered in NPR is accurate and consistent with diagnoses received from diagnostic interviews [26]. Finally, the analyses would benefit from a longer period of investigation, as the NRPHC is only available from 2017 [8].

## Conclusions

This investigation of diagnostic information in NRPHC LTC found that the completeness was low, but that diagnostic information could be extracted from other sources (NRPHC GP and NPR).
